# Image Imputation with conditional generative adversarial networks captures clinically relevant imaging features on computed tomography

**DOI:** 10.1371/journal.pdig.0000970

**Published:** 2025-08-13

**Authors:** Joseph Rich, Jonathan Le, Ragheb Raad, Tapas Tejura, Ali Rastegarpour, Inderbir Gill, Vinay Duddalwar, Assad Oberai

**Affiliations:** 1 Department of Radiology, University of Southern California, Los Angeles, California, United States of America; 2 Department of Biology and Bioengineering, California Institute of Technology, Pasadena, California, United States of America; 3 Department of Aerospace & Mechanical Engineering, University of Southern California, Los Angeles, California, United States of America; 4 Department of Radiology, Los Angeles General Medical Center, Los Angeles, California, United States of America; 5 Institute of Urology, University of Southern California, Los Angeles, California, United States of America; 6 Radiomics Lab, University of Southern California, Los Angeles, California, United States of America; 7 Alfred E Mann Department of Biomedical Engineering, University of Southern California, Los Angeles, California, United States of America; University of Florida, UNITED STATES OF AMERICA

## Abstract

Kidney cancer is among the top 10 most common malignancies in adults, and is commonly evaluated with four-phase computed tomography (CT) imaging. However, the presence of missing or corrupted images remains a significant problem in medical imaging that impairs the detection, diagnosis, and treatment planning of kidney cancer. Deep learning approaches through conditional generative adversarial networks (cGANs) have recently shown technical promise in the task of imputing missing imaging data from these four-phase studies. In this study, we explored the clinical utility of these imputed images. We utilized a cGAN trained on 333 patients, with the task of the cGAN being to impute the image of any phase given the other three phases. We tested the clinical utility on the imputed images of the 37 patients in the test set by manually extracting 21 clinically relevant imaging features and comparing them to their ground truth counterpart. All 13 categorical clinical features had greater than 85% agreement rate between true images and their imputed counterparts. This high accuracy is maintained when stratifying across imaging phases. Imputed images also show good agreement with true images in select radiomic features including mean intensity and enhancement. Imputed images possess the features characteristic of benign or malignant diagnosis at an equivalent rate to true images. In conclusion, imputed images from cGANs have large potential for clinical use due to their ability to retain clinically relevant qualitative and quantitative features.

## Author summary

Kidney cancer is one of the most common cancers in adults, and doctors often use a special type of CT scan that captures four different phases to detect and understand the disease. However, some of these scans can be missing or unusable due to technical problems, which can affect diagnosis and treatment planning. To address this, we used a type of artificial intelligence called a conditional generative adversarial network (cGAN) to “fill in” missing scans by learning from complete ones. After training our model on data from 333 patients, we tested it on 37 new patients to see how well the AI-generated scans matched the real ones. We compared 21 important imaging features and found that the AI-generated scans closely matched the originals, with over 85% agreement on all 13 key categorical features. These generated images also preserved important tumor characteristics and image details. Our findings suggest that AI can help recover missing medical images in a way that still supports accurate clinical decision-making. This technology could improve care for kidney cancer patients and reduce the impact of incomplete imaging.

## Introduction

Kidney cancer is among the top 10 most common malignancies in adults. Diagnosis and monitoring of renal masses is conducted with multiphase contrast-enhanced computed tomography (CECT) imaging, which usually images the mass at four points based on the point of contrast enhancement: precontrast (before contrast injection), corticomedullary/arterial (30–40 seconds after contrast injection), nephrographic/venous (100 seconds after contrast injection), and excretory/urographic (5–10 minutes after contrast injection) [1]. The precontrast phase is imaged before contrast, and serves as a useful baseline for the organ’s natural appearance. The corticomedullary phase depicts peak contrast flow in the renal cortex, useful for visualizing anomalies with these structures. The nephrographic (venous) phase is imaged at the point of peak contrast flow through both the cortex and medulla, and is typically best for visualization of renal masses components. The excretory phase depicts the kidney after processing the contrast, and is useful for evaluating the functionality of the excretory system (i.e., renal pelvis, ureters, bladder, urinary tract).

The most important determination in renal mass imaging workup is the distinction between a benign vs. malignant mass. This distinction is commonly made by assessing a range of clinically relevant features from this four-phase imaging study. These features can be qualitative, including tumor shape, location, heterogeneity, or margin definition; or quantitative, including intensity, enhancement, and size. However, the distinction between benign and malignant tumors is often not clear from imaging features alone with a significant error rate up to 30% [2], justifying the search for a better way to make the diagnosis [3–7].

Missing or poor quality data is a common occurrence in multi-phase renal imaging that can reduce the diagnostic accuracy of radiologists. Imaging data can be lost or corrupted for a multitude of reasons. For instance, images can be lost due to the lack of interoperability between different imaging protocols and vendors of imaging equipment across medical institutions [8]. Additionally, the quality of CT images can be degraded by artifacts such as motion or blurring [9]. The absence of any of the four phases has been shown to reduce the accuracy of radiologic diagnosis and radiomic characterization of renal masses, as distinguishing between benign and malignant tumors relies on subtle differences on visual inspection of the four CECT phase images [10]. Moreover, loss of any of these images can impede access to important anatomical information that is critical for surgical planning in cases where surgical removal of the mass is necessary. However, recovering missing phase data would be costly, costing $449 per abdominal CT scan with contrast on average [11] as well as significant added time from both the patient and healthcare providers. Image imputation, which is the process of generating missing images given available images, shows potential to fill in missing phase data in multi-phase renal imaging given images from the remaining three phases.

Deep learning approaches through generative adversarial networks (GANs) have recently shown promise in this image imputation task. One study designed a probabilistic deep generative model designed to impute missing phase images in CECT imaging, incorporating quantified uncertainty and a novel style-based adversarial loss to enable better-informed medical decisions [12]. Another study introduced a novel probabilistic deep-learning algorithm using a GAN trained on CECT images to infer and sample the probability distribution of missing images with enhanced reliability via a unique style loss and Bayesian inference [13]. Most recently, a conditional GAN (cGAN) was trained on the task of image imputation of four-phase CECT renal imaging [14]. This cGAN model yielded good technical accuracy on the metrics of Structural Similarity Index Measure (SSIM), Peak Signal-to-Noise Ratio (PSNR) and Normalized Mean Square Error (NMSE), indicating better performance compared to related model architectures. This model also performed well on a range of automatically-extracted radiomic metrics [14]. The clinical utility of these results, however, was not explored.

The purpose of this study was to determine the clinical utility of imputed computed tomography (CT) scans from this cGAN by focusing the evaluation of the images on clinically-relevant features.

## Materials and methods

### Ethics statement

Data for this study was extracted from an Institutional Review Board (IRB) approved Kidney Mass Data and Specimen Collection project. Informed consent for the repository was obtained by the University of Southern California (USC) IRB consistent with §45 CFR 46.116(f). The study was conducted in accordance with USC policies, IRB policies, and federal regulations. Subject privacy and confidentiality were protected according to applicable HIPAA, and USC IRB policies and procedures.

### Study design

**Fig 1:**
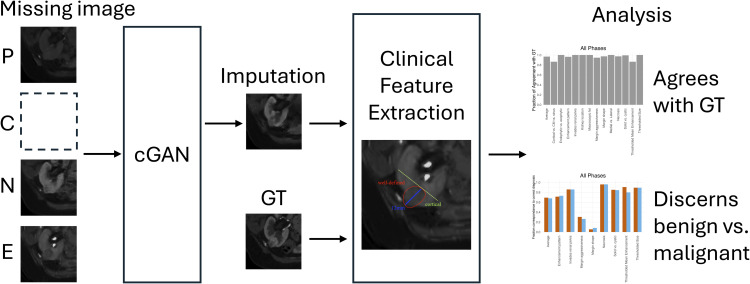
Project overview schematic. Each image is imputed by inputting the other three phases into the cGAN model, and both the imputed image and its ground truth counterpart are passed through the clinical feature extraction pipeline. Analysis includes comparing the clinical similarity of the imputed images to their ground truth, as well as comparing each of their abilities to correctly distinguish benign and malignant masses.

The study design is described in Fig 1. The study included 37 preoperative four-phase renal CT imaging studies obtained from 35 patients with renal masses (imputation was done twice on two patients with different crops). Each study consisted of four phases (non-contrast, corticomedullary, nephrographic, and excretory), yielding a total of 148 images. For each image, the remaining three phases within the same study were used to impute the held-out image, resulting in 148 imputed images. The cGAN was trained and validated with an additional 333 patient studies (1332 images) training set collected from the same cohort, with images imputed with the same process as described earlier. The architecture is described fully by Raad et al. in Fig 6 [14]. Briefly, the generator in this architecture employs a U-Net structure with conditional instance normalization (CIN) at every scale of its contracting and expanding branches, introducing stochasticity and dimension flexibility while utilizing dense blocks instead of residual blocks to optimize performance and reduce trainable parameters. The U-net is formed of an encoder phase and a decoder phase. The encoder reduces the size of the input tensor to extract the image features while the decoder takes that result and up-samples until an image of the same size as the input tensor is generated. The critic’s architecture consists of dense block-based down-sampling followed by a fully connected network, which outputs a scalar value. The critic uses layer normalization instead of CIN since the latent variable is not evaluated, simplifying the critic’s design compared to previous more complex models. The generator outputs a prediction of the missing image using the other three while the critic is trying to distinguish fake generated images from real images. The goal at the end of training is for the generator to be able to fool the critic into believing that the generated images are real. Each image was augmented through rotations, cropped to a 128 x 128 pixel size to focus on the tumor region, and further annotated by two radiologists to select circular regions of interest (ROIs) on the tumor. The augmented images were rotations of 10, 20, and 30 degrees in both clockwise and counterclockwise directions. Images with small tumors were also augmented through shifts in the four directions.

A list of 21 clinically relevant imaging features was created in consultation with radiologists and the literature [15]. There were 13 categorical features as follows:

1. Enhancement pattern (homogeneous vs. heterogeneous):
  a. Homogeneous: even contrast enhancement throughout the lesion.
  b. Heterogeneous: uneven or patchy enhancement, possibly due to necrosis or differing tissue types.
2. Margin shape (smooth vs. lobulated)
  a. Smooth: rounded, uniform borders.
  b. Lobulated: irregular or scalloped edges with lobes.
3. Margin aggressiveness (well-marginated/visible vs infiltrative)
  a. Well-marginated/visible: sharply defined tumor borders.
  b. Infiltrative: ill-defined borders suggesting invasion into surrounding tissue.
4. Endophytic vs. exophytic (endophytic, < 50% exophytic, 50/50, > 50% exophytic)
  a. Endophytic: located primarily within the kidney.
  b. Exophytic: proportion projecting outward from the kidney.
5. Solid vs cystic
  a. Solid: composed primarily of tissue.
  b. Cystic: containing fluid-filled areas.
6. Area of necrosis/hypoattenuation (present vs absent): whether or not there are visible non-enhancing regions within the lesion suggestive of necrosis
7. Macroscopic fat (present vs absent). whether or not there is visible fat within the lesion on CT.
8. Kidney location (right vs. left): side of the body the tumor is located on.
9. Medial vs lateral
  a. Medial: near the renal hilum.
  b. Laterial: toward the outer convex edge of the kidney.
10. Cortical vs corticomedullary vs sinus location
  a. Cortical: outer region of the kidney.
  b. Corticomedullary: junction of cortex and medulla.
  c. Sinus: central fat-filled region containing vessels and collecting system.
11. Invasion of renal pelvis (yes/no): whether the tumor extends into the renal pelvis/collecting system.
12. Thresholded mean enhancement (threshold of 10 HU): binary feature indicating if mean enhancement exceeds 10 Hounsfield Units (HU)
13. Thresholded size (threshold of 10 mm): binary feature indicating if the lesion size exceeds 10 millimeters.

There were 8 quantitative continuous features as follows:

1. Lesion size: longest tumor dimension in any plane (mm).
2. Lesion size (2nd dimension): perpendicular diameter to the longest axis (mm).
3. Min ROI intensity
4. Mean ROI intensity
5. Max ROI intensity
6. Min ROI enhancement
7. Mean ROI enhancement
8. Max ROI enhancement

These features were manually extracted from all ground truth (GT) and imputed images. Most categorical features were extracted through visual assessment under the guidance of a radiologist (S1 Table). The values for these 21 clinically relevant features for each of the 148 images, for both GT and imputed images, can be found in S1 Table. Thresholded intensity and size features were derived from their quantitative measurements. ROI intensity and enhancement metrics were calculated by restricting the image to a range of [-150, 1000] Hounsfield Units (HU) and measuring the statistic on a numpy array storing pixel values. Enhancement was defined as the pixel-wise difference of the precontrast phase from any of the latter three phases. Lesion size was measured manually with a matplotlib graphical user interface (GUI).

For assessing diagnostic accuracy of discerning between benign vs. malignant status, we selected a subset of our categorical features and some thresholded quantative features that are established to be characteristic of benign or malignant tumors in clinical practice and literature [3,6]. We mapped the following 6 features to benign diagnosis and 8 features to malignant diagnosis:

Benign:

1. Homogeneous enhancement pattern
2. Smooth margin shape
3. Well-defined margin aggressiveness
4. Cystic appearance
5. Small size <10 mm [16]
6. Mean enhancement <10 HU

Malignant:

1. Heterogeneous enhancement pattern
2. Necrosis
3. Lobulated margin shape
4. Infiltrative margin aggressiveness
5. Solid appearance
6. Invades renal pelvis
7. Large size >10 mm
8. Mean enhancement >10 HU

To quantify hallucination of the model, we used the Hallucination Index from MICCAI [17]. This index considers hallucination to be the noise added specifically by a machine learning model (i.e., variance that cannot be explained by noise). We converted the tumor region of each imputed image and real image to a histogram of pixel values to approximate the probability distribution representing each image. We then calculated the Hellinger Distance between each pair of images, with 0 representing identical distributions (no hallucination) and 1 representing no overlap between distributions (much hallucination).

For an additional analysis of overall image resemblance between the true and imputed image, three experienced abdominal radiologists labeled each imputed image on a scale of 1–5 to assess similarity and likelihood to their true image, with 1 representing high dissimilarity and 5 representing high similarity. Each radiologist was blinded on the label of each image as imputed vs. true. The assigned score was representative of the usability of the imputed image in a clinical context compared to having missing data.

Data analysis was performed in R with base R and the tidyverse packages (dplyr for data manipulation, ggplot2 for all plots). Statistical significance was assessed with a variety of tests. For average per-feature agreement of categorical clinical features between imputed and GT images, the binomial test was used. For the comparison of the diagnostic accuracy of imputed images to GT on benign vs. malignant, the two proportion z-test was used. For the comparison of mean standard deviation between images where the features agreed with GT vs. those which did not, a Wilcoxon rank sum test with Benjamini-Hochberg multiple testing correction was used. Inter-rater variability for the evaluation of the three radiologists was calculated with weighted kappa value and Prevalence- and Bias-Adjusted Kappa (PABAK) calculation.

## Results

Patient demographics are described in Table 1. The majority of the patient population is male at 70.3%, and the mean age is 57.7 years old. Caucasians and hispanic origin were the most frequent subgroups. Cases were generally lower grade with a mean grade of 2, but 89% of cases were diagnosed as malignant.

**Table 1:** Demographics of patient cohort.

Patient demographics	Statistic (n = 37)
Gender	Male: 70.3%
	Female: 29.7%
Age (mean ± SD)	57.7 ± 12.1
Race	White: 67.6%
	Hispanic: 19.9%
	Asian: 8.1%
	Black: 2.7%
	Unknown: 2.7%
Grade (mean ± SD)	2.2 ± 0.4
Malignancy	Benign: 10.8%
	Malignant: 89.2%
Aggressiveness	Aggressive: 40.5%
	Non-aggressive: 59.5%

**Fig 2:**
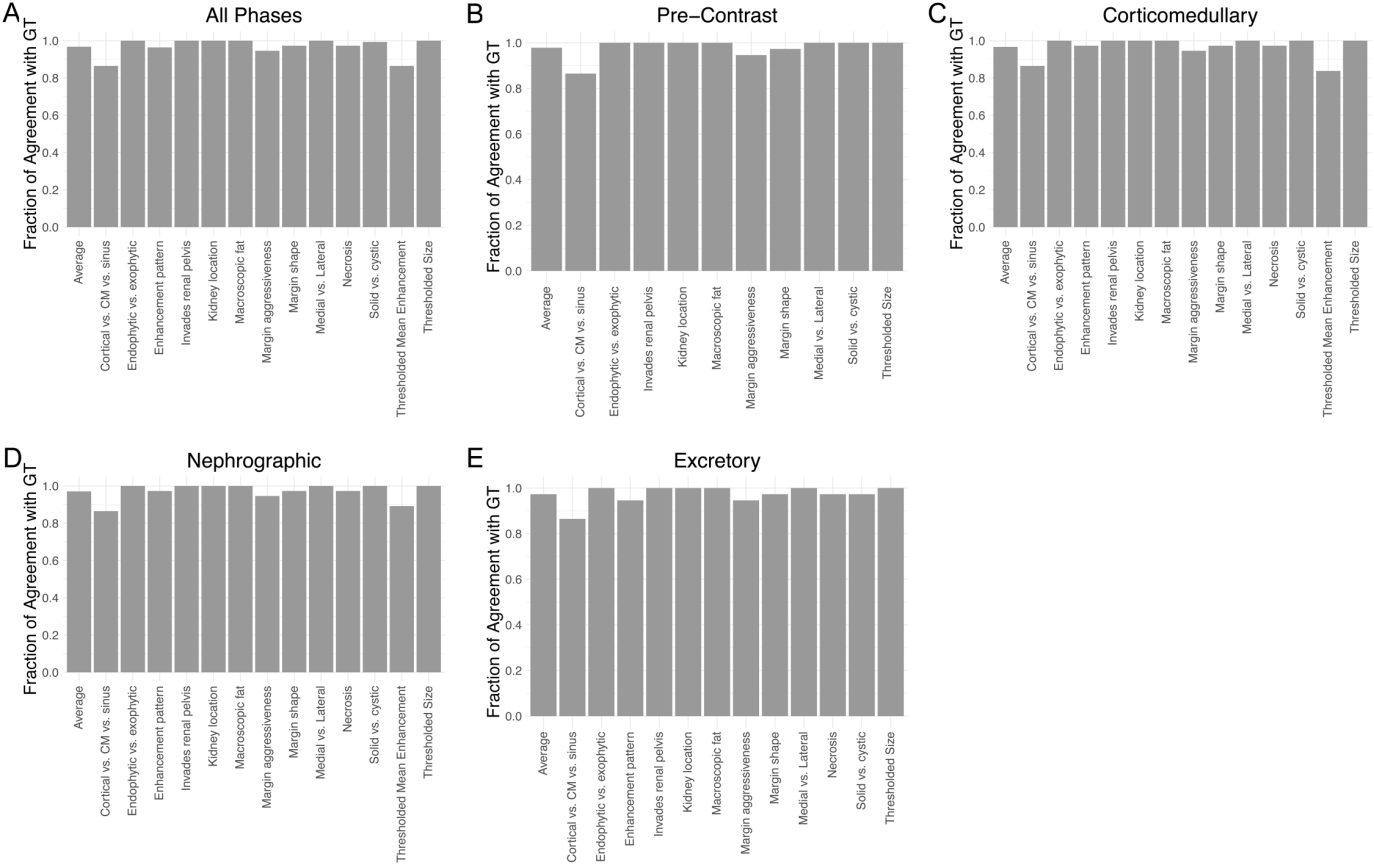
Imputed images agree with ground truth (GT) on categorical features. Bars show the fraction of images which agree with its corresponding image in the GT per feature. (A) All phases, (B) Pre-Contrast Phase, (C) Corticomedullary Phase, (D) Nephrographic phase, (E) Excretory phase.

All 13 categorical clinical features had greater than 85% agreement rate between true images and their imputed counterparts, with most features showing above 95% agreement (Fig 2A). This high accuracy is maintained when stratifying across imaging phases (Fig 2B-E). The features which show the lowest agreement with GT are cortical vs. corticomedullary vs. sinus, thresholded mean enhancement, and margin aggressiveness. A heatmap depicting qualitative feature agreement for each image across each figure indicates that feature disagreement was often consistent among all phases within a given imaging study, and that no single imaging study contained more than two features that disagreed with their ground truth (S1 Fig).

**Fig 3:**
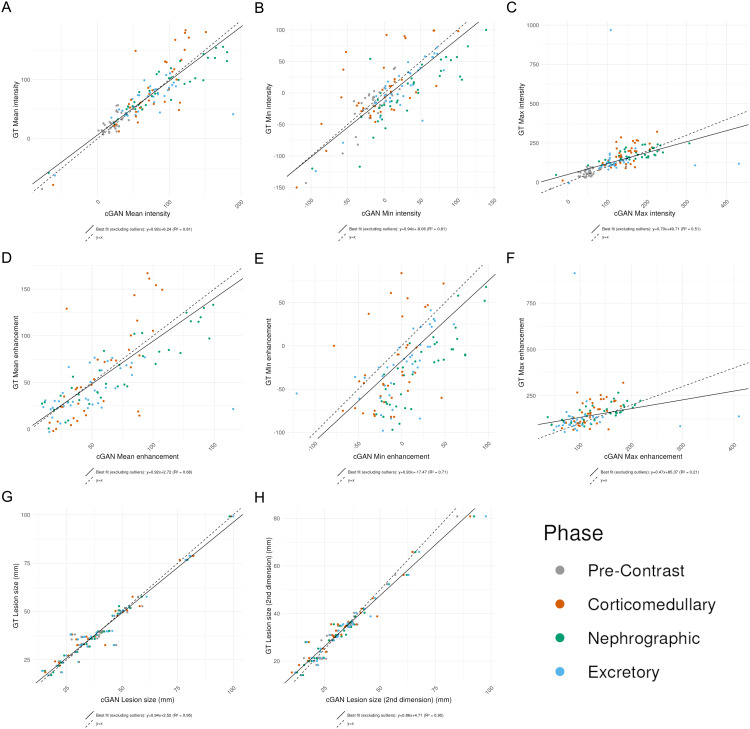
Imputed images correlate well with their respective ground truth (GT) images on multiple clinically-relevant quantitative features. (A) Mean intensity, (B) Min intensity, (C) Max intensity, (D) Mean enhancement, (E) Min enhancement, (F) Max enhancement, (G) Lesion size, (H) Lesion size (2nd dimension). Colors = phases; solid line = best fit line; dashed line = y = x.

The 8 quantitative clinical features also generally show good agreement between true images and their imputed counterparts. In particular, min intensity, mean intensity, and lesion size in both dimensions show a high correlation with R^2^ > 0.9 (Fig 3). However, max intensity, mean enhancement, and min enhancement show a more modest agreement with R^2^ between 0.5 and 0.7 (Fig 3).

**Fig 4:**
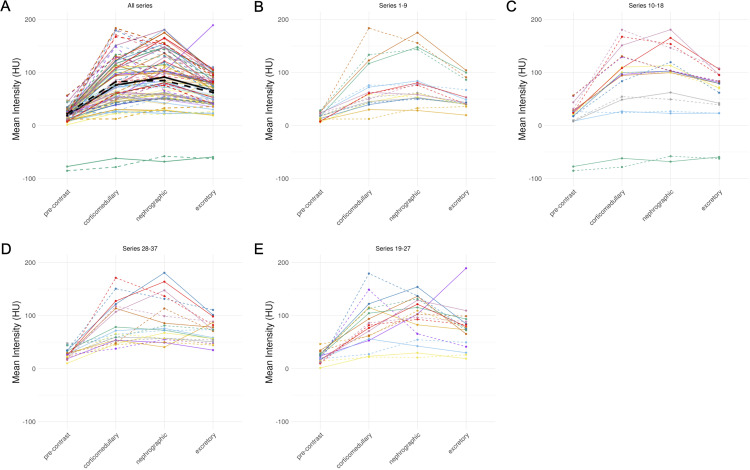
Imputed images show similar phase intensity diagrams to their ground truth (GT) counterparts. (A) All 37 imaging studies, split into groups of 9-10 (B-E) for visualization. Color, alpha = imaging study; solid = imputed image; dashed = GT for the corresponding imputed image; Black lines = average values.

One of the applications of mean ROI intensity is in the construction of a four-phase intensity plot. The values and shape of the line segment are diagnostic for distinguishing clear cell renal cell carcinoma, papillary renal cell carcinoma, oncocytoma, and chromophobe renal cell carcinoma and angiomyolipoma (5). The intensity plots by connecting the GT images were overall similar to those created by connecting imputed images (Fig 4). While the mean magnitude difference between GT and imputed image was 15.72 (8.22 for precontrast, 23.5 for corticomedullary, 16.0 for nephrographic, 15.2 for excretory), these generally balanced out, as the mean (signed) difference between GT and imputed image was -0.01 (-3.77 for precontrast, -6.53 for corticomedullary, 6.44 for nephrographic, 3.80 for excretory) (Fig 4A, black line).

**Fig 5:**
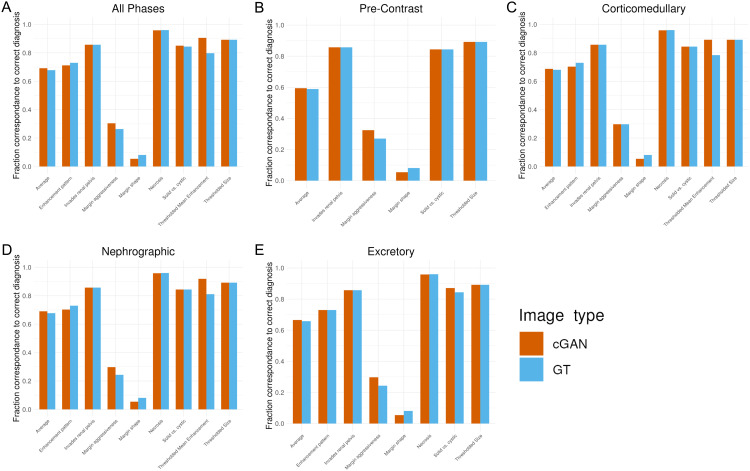
Imputed images map clinical features characteristic of benign vs. malignant diagnosis status at a level similar to ground truth (GT). Bars show the fraction of images which contain the clinical feature that corresponds to the correct diagnosis. (A) All phases, (B) Pre-Contrast Phase, (C) Corticomedullary Phase, (D) Nephrographic phase, (E) Excretory phase.

In addition to the imputed images showing good agreement with GT on clinically relevant features, it also maintained the features characteristic for diagnosing between benign and malignant masses. On average, all features are represented at equal rates between imputed images and GT for each feature, including when stratifying by phase (Fig 5).

**Fig 6:**
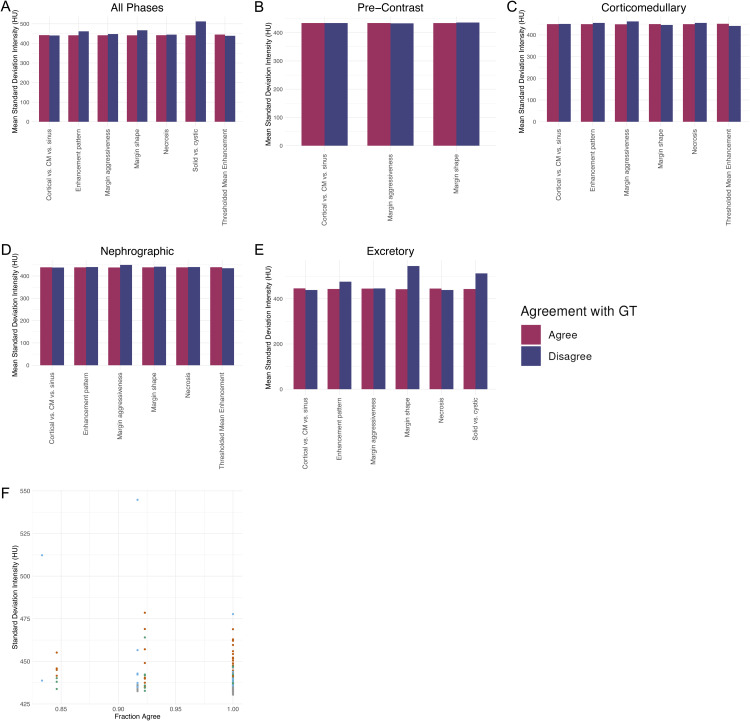
Standard deviation of imputed images does not correlate with agreement with ground truth (GT). (A) All phases. (B) Pre-Contrast Phase. (C) Corticomedullary Phase. (D) Nephrographic phase. (E) Excretory phase. (F) Relationship between standard deviation and the fraction of features in agreement with GT per image.

For each feature, the set of 148 imputed images was divided into the set in which the imputed image agreed with the GT and the set in which it did not, and the mean standard deviation of the imputed images in each group was compared. There was no statistical difference between the mean standard deviation between these groups for any feature, including when stratifying by phase (Fig 6A-E). In addition to calculating the predictive value of standard deviation on a per-feature basis, we calculated the predictive value of standard deviation on a per-image basis. When binning imputed images by the fraction of categorical features which agreed with GT, there was no significant correlation between fraction of agreeing features and mean standard deviation of the images in each bin (Fig 6F).

**Fig 7:**
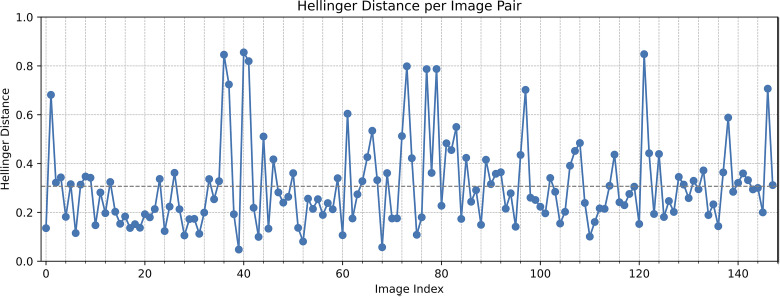
Hallucination Indices for each of the 148 analyzed images. Gray dashed line = mean (0.3069).

The Hallucination Index had a mean of 0.307 and a variance of 0.030 (Fig 7). Inspection of some of the images with a value well above the average indicate a trend that, while pixel intensity values and heterogeneity differ between the true and imputed image, there is no appreciable difference in many other aspects regarding tumor appearance (S2 Fig).

**Fig 8:**
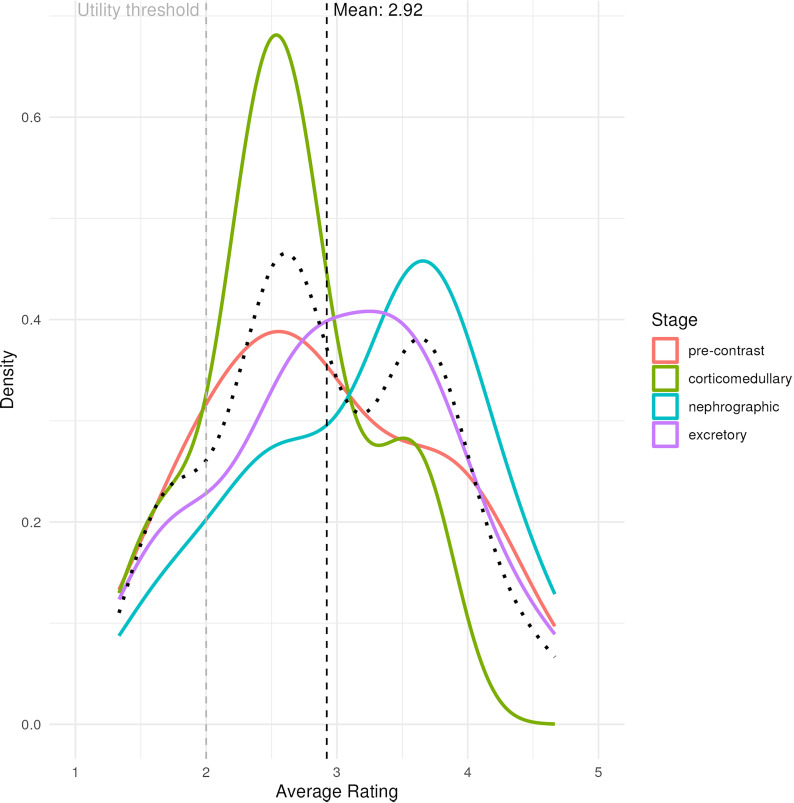
Smooth density plot of radiologist evaluation of similarity between imputed and true images on 1-5 scale. Black curve = average of four phases; Black dashed line = average score; Gray dashed line = thresholded value for clinical utility and inter-rater variability calculations.

Radiologist evaluation demonstrated that most imputed images provided clinical utility when compared to having no data, with only 14% of images falling below a score of two that would indicate dissimilarity to the ground truth. The mean score of all images was 2.92, with no statistical significance by image phase (Fig 8). Inter-rater agreement varied across reader pairs, with total agreement ranging from 55.6% to 82.2%. Weighted kappa values ranged from 0.07 to 0.52, indicating poor to moderate agreement, while PABAK values were consistently higher (0.33 to 0.73), reflecting the influence of prevalence and bias on standard kappa estimates. (Table 2).

**Table 2:** Radiologist inter-observer variability on overall similarity of imputed and true image. R1, = Review 1; R2 = Reviewer 2; Kappa = Cohens’ Kappa; PABAK = Prevalence- and Bias-Adjusted Kappa; Rad = Radiologist; CI = Confidence Interval.

R1	R2	Kappa	PABAK	% Agree 0	% Agree 1	% Agree total
Rad1	Rad2	0.52 95% CI (0.34, 0.7)	0.73 95% CI (0.63, 0.84)	14.95%	67.29%	82.24%
Rad1	Rad3	0.39 95% CI (0.24, 0.54)	0.58 95% CI (0.45, 0.71)	16.82%	55.14%	71.96%
Rad2	Rad3	0.07 95% CI (-0.11, 0.25)	0.33 95% CI (0.19, 0.47)	14.81%	40.74%	55.56%

## Discussion

These results demonstrate the potential clinical utility of imputed images generated by a cGAN in four-phase renal CECT studies. These imputed images captured the clinically relevant features of renal masses at a high rate across all phases, including both categorical and quantitative features (Fig 3). As a result, these imputed images have the potential to serve as a substitute for missing or corrupted images in multi-phase renal imaging. Since differentiating between a benign and malignant mass relies on subtle differences on visual inspection of CT imaging, having access to additional phases would enhance the diagnostic accuracy of radiologists while also reducing medical costs and patient exposure to radiation [18].

The quality of imputed images out of the multiphase imputation task can be divided into four categories - maintaining what is retained in the other 3 phases, omitting what is not retained from the other 3 phases, adding what should be added compared to the other 3 phases, and not adding what should not be added. The imputed images in this study show excellent performance in all of these tasks, which are all reflected in the percentage of agreement (Fig 2).

Regarding the comparison of diagnostic accuracy, we focused on features characteristic of benign vs. malignant as a means of ensuring that all essential features were present and not being hallucinated. Benign and malignant renal masses have a wide range of possible appearances across patients, so all qualitative features are considered together by the radiologist when determining the diagnosis. Part of the diagnostic process involves roughly determining the proportion of features present which are characteristic of benign vs. malignant cases. In this sense, the imputed images predict the correct diagnosis at a rate equal to GT.

We highlight two representative examples to illustrate the strengths and limitations of the imputation approach. In the first case, the imputed image demonstrates strong alignment with the ground truth across key clinical features, including margination, exophytic nature, mean ROI intensity, and enhancement pattern, suggesting accurate recovery of clinically relevant characteristics. In contrast, the second example shows partial agreement—capturing features such as the partially exophytic nature and cortical location—while diverging in others. Specifically, the imputed image exhibits a higher mean ROI intensity, less well-defined margination, and a more homogeneous enhancement pattern compared to the ground truth, which presents with a lower mean intensity, sharper margins, and greater heterogeneity. These examples underscore both the potential and current limitations of the model in preserving nuanced radiologic features during imputation.

Although no association was found between standard deviation and clinical features in the imputed images, this is likely driven by the fact that the imputed images performed so well. There were relatively few cases of features disagreeing between the imputed images and GT, thus leading to small group sizes when stratifying by agreement per-feature. And even the worst-performing imputed images still had greater than 80% agreement with GT, limiting the range to assess correlation (Fig 6F).

One of the potential reasons for the lower level of agreement between imputed and GT images for margin definition and smoothness is that the averaging across all cGAN images smoothens out the appearance of the tumor in some cases due to slight alterations in locations across different predictions. Similarly, there may have been a lower level of agreement in the distinction between cortical vs. corticomedullary vs. sinus mass location because each phase depicts different parts of the kidney more clearly. Thus, when generating the image for a missing phase using the other three phases, the cGAN image may not capture the anatomical details of that phase as clearly. However, when utilizing the imputed image in conjunction with the other three phases to visually analyze the mass, as is done in clinical practice, the anatomical distinctions become clearer.

We analyze a combination of categorical and quantitative features, as both types of features are important in clinical decision-making. Quantitative features such as tumor size and intensity provide measures in a continuous scale that allows for nuanced decision-making. Categorical features such as tumor appearance and margins can convert measures that are difficult to quantify (such as a complex shape) or where thresholded categories are more insightful than continuous differences (such as size thresholds) into groups that can be generalized and studied for their diagnostic and predictive power. The fact that our model performs very well on categorical features indicates that the observed differences in quantitative feature prediction generally are not substantial in size and do not cause an imputed image to provide qualitatively incorrect information to the radiologist.

We stratify a subset of our features based on those that hold strong predictive power for identifying a lesion as benign or malignant based in the literature. While the diagnosis of a renal mass as benign vs. malignant relies on a combination of features and the intuition and experience of a radiologist, we identify these categories because they represent qualitative image characteristics that play a substantial role in a radiologist’s evaluation. Although no single feature alone is sufficient for this diagnosis, this categorization allowed us to confirm that the imputed images were generally not altering these core qualitative properties of a scan.

We selected a conditional generative adversarial network (cGAN) architecture for imputing missing phase data in 4-phase renal CT imaging due to its ability to learn structured, paired transformations between modalities while explicitly conditioning the generation process on available phases. Unlike traditional GANs, which generate data from noise without context, cGANs incorporate known input information—such as the non-missing imaging phases—to guide the synthesis of missing data. Diffusion models, an alternative class of generative models, iteratively denoise random noise through a learned reverse process, often achieving high image fidelity but requiring hundreds of computational steps per sample [19]. Compared to diffusion models, cGANs are significantly faster at inference due to their single-pass generation and offer more direct control via conditioning inputs [20]. Additionally, GANs have been established in the medical imaging community for a longer time, with well-understood training dynamics and a wide range of successful applications, making cGANs a more practical and interpretable choice for clinical imaging tasks at the time this study was conducted.

A limitation of this study is the small sample size and use of a single cohort for both model training and testing. However, the strong performance of the model outputs demonstrates a proof-of-concept for the clinical utility of the methodology, and future efforts can include scaling of the size and heterogeneity of datasets involved. Additional future directions could include stratification of performance by demographic factors, ability to correctly diagnose a wider variety of conditions (e.g., grade, stage, renal cell carcinoma subtypes), and use of these imaging data into further predictive models for diagnosis, prognosis, and treatment planning. Another limitation of the study is the use of a single annotator for the qualitative radiologic features. While multiple radiogists and students were involved in producing these annotations, future efforts could involve gathering multiple independent annotations and determing inter-rater agreement.

## Conclusion

Imputed images in four-phase renal CECT studies generated by this cGAN are highly similar to their true counterparts in maintaining their clinically relevant features. This similarity is observed both for qualitative and quantitative features, and across all phases. They could serve a role in enhancing diagnostic accuracy of radiologists, saving imaging costs in a clinical setting, and providing high quality radiologic images that could improve medical research and education.

## Supporting information

S1 Fig Heatmap of agreement for each categorical feature by each pair of real-imputed images. Light blue = feature agreement; dark blue = feature disagreement.
(TIFF)

S2 Fig A representative pair of images with above-average Hallucination Index. Red outline = tumor boundary.
(TIFF)

S1 Table Analysis of patient image information across the clinical features of interest for the ground truth and cGAN-generated images. (XLSX)
