# Supplementary figures and images for: Image Imputation with conditional generative adversarial networks captures clinically relevant imaging features on computed tomography
Source: PLOS Digit Health. 2025 Aug 13;4(8):e0000970. doi: 10.1371/journal.pdig.0000970 (PMC12349720; doi:10.1371/journal.pdig.0000970)

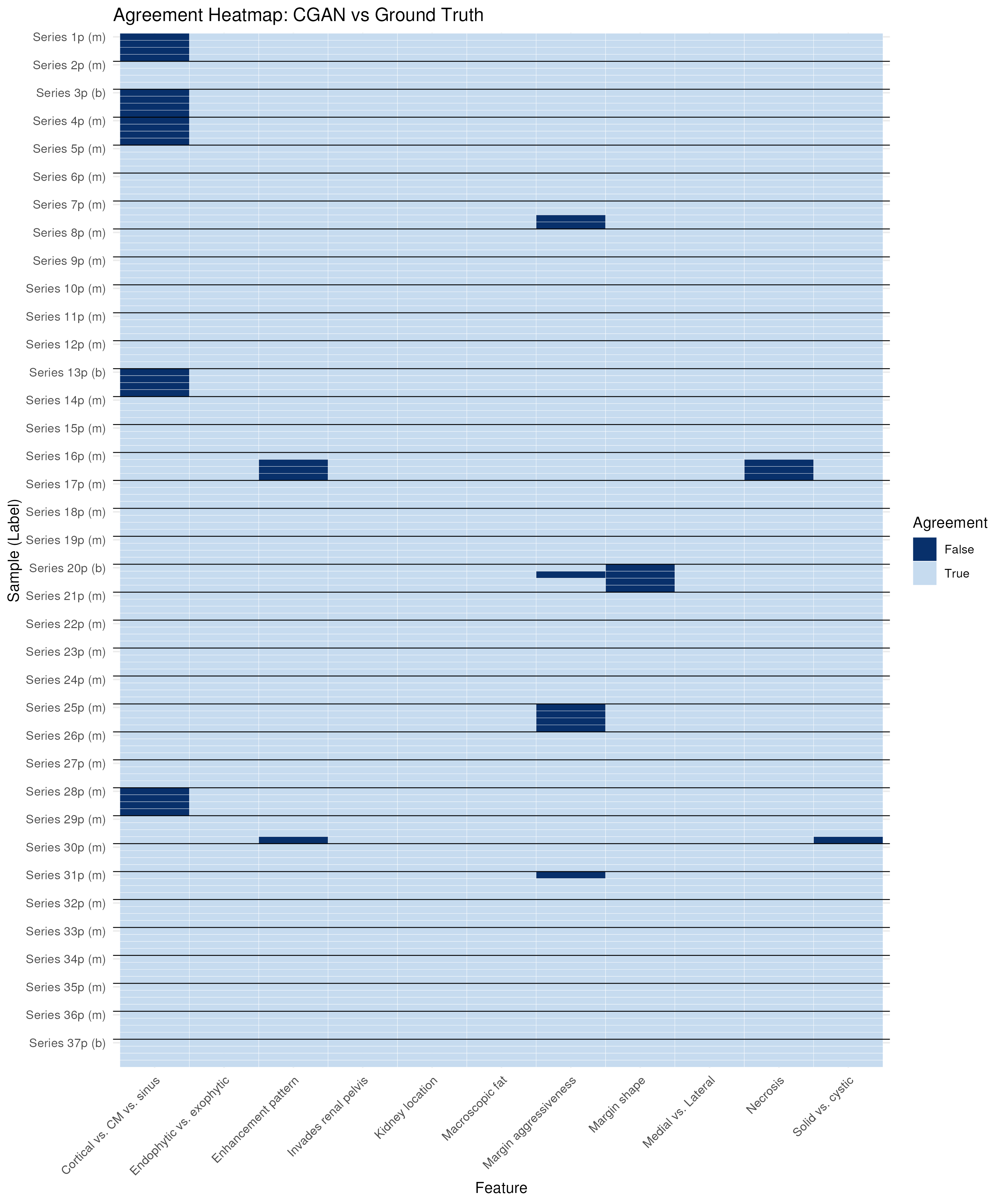

Supplement: S1 Fig — Light blue = feature agreement; dark blue = feature disagreement. (TIFF) [file pdig.0000970.s001.tiff]

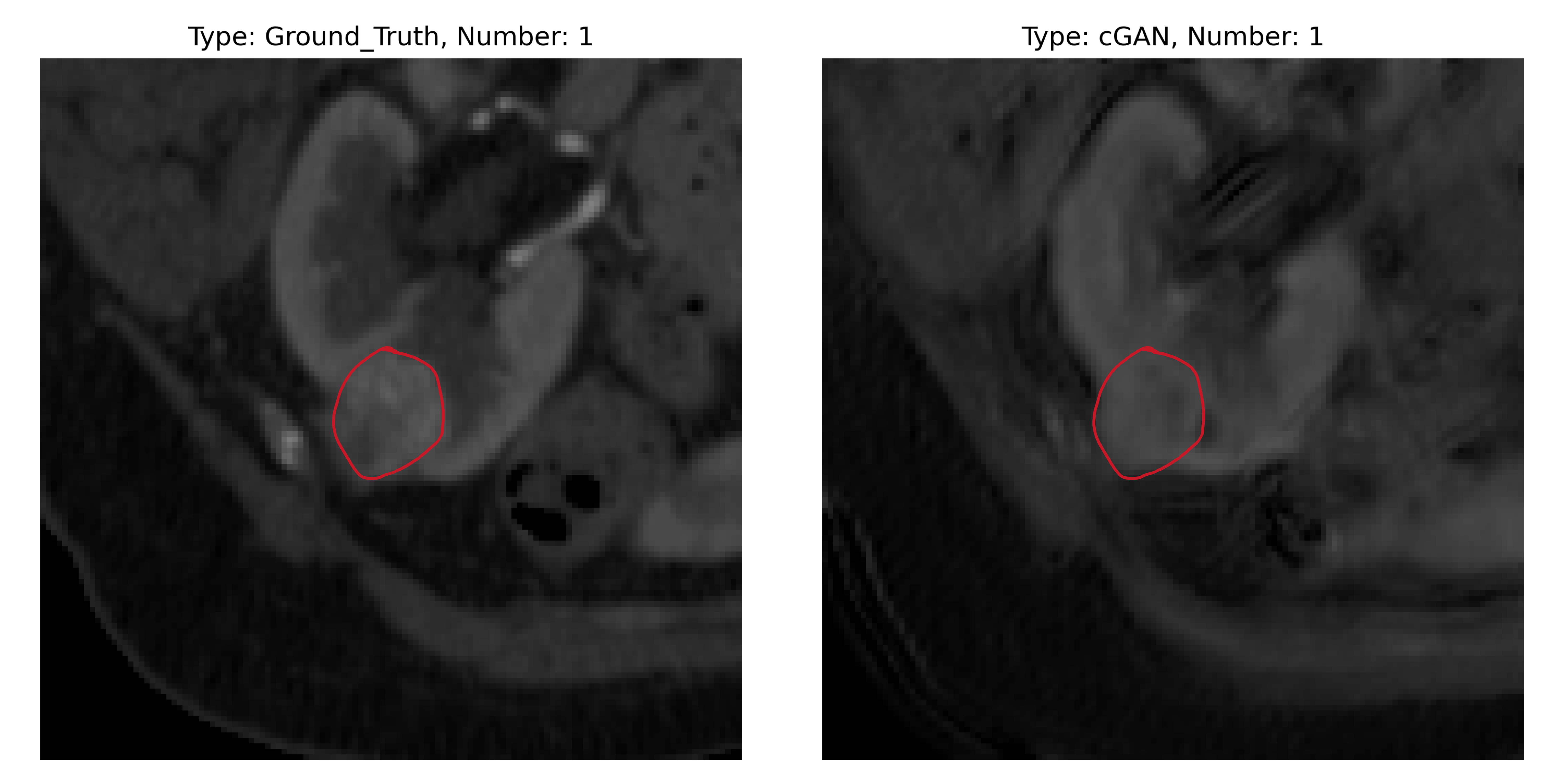

Supplement: S2 Fig — Red outline = tumor boundary. (TIFF) [file pdig.0000970.s002.tiff]
